# Rapid Maxillary Expansion for Obstructive Sleep Apnea among children - Systematic Review and Meta-analysis

**DOI:** 10.5935/1984-0063.20190123

**Published:** 2020

**Authors:** Sarah Ahmed Bahammam

**Affiliations:** Taibah University, Pediatric Dentistry and Orthodontics - Medina - Saudi Arabia.

**Keywords:** Apnea Hypopnea Index, Sleep Apnea, Obstructive Sleep Apnea

## Abstract

A systematic review and meta-analysis is conducted for children with obstructive sleep apnea (OSA) treated with rapid maxillary expansion (RME). The study systematically and independently reviewed extracted articles from 2009 to 2019. The quality evaluation and selection of these studies was based on the high quality of NICE criteria. EMBASE, Scopus and other five databases were used to extract international publications. The findings indicated that the apnea-hypopnea index was enhanced after rapid maxillary expansion among children with OSA. It revealed that apnea hypopnea index enhances to 73% in children with obstructive sleep apnea after ≤ three years follow-up, while it was 77% in children with obstructive sleep apnea after > 3 years. The articles included in this meta-analysis reported differential outcomes based on different inclusion or exclusion criteria and diverse patient populations. Critical evaluation of previous literature suggests diagnosing the disorder at an early stage for reducing the adverse health outcomes and formulating an appropriate treatment plan.

## INTRODUCTION

Obstructive sleep apnea (OSA) is a significant disorder in sleep disorders wild mild to moderate symptoms^1^^,^^2^. Villa et al.^3^ reported its prevalence from 1% to 5.7% in children, which is due to the prominence of lymphoid tissue at this age^4^. Its etiological symptoms include: snoring, increased daytime somnolence, and different behavioral and neurological repercussions^5^. Various abnormalities cause the pathogenesis of OSA syndrome, i.e., larger craniocervical angulation, retrognathia of maxilla and mandible, elongated soft palate, increased lower facial height, inferior positioned hyoid bone and decreased posterior airway space^6^^-^8. Other conditions leading to sleep apnea include hypotonia, nasal obstruction, obesity and macroglossia.

OSA narrows the upper airway, which is derived from the narrow and long-faced, maxillary constriction or high arched palates along with some extent of mandibular retrusion^7^. Despite the improvement in the mandibular correction and maxillomandibular anomalies, the craniofacial and orthodontic abnormalities in the children with OSA have been neglected^7^.

Various therapies for OSA include positive airway pressure devices, nasal devices, myofunctional therapy, oral appliances and sleep surgeries^8^. Rapid maxillary expansion (RME) is also found to be effective for the treatment of dental crowding and malocclusion, and for high arched or narrow hard palates, all leading to OSA^8^. RME is applied through orthodontic appliances, in which pressure is directly applied to the maxillary suture via anchor teeth^7^. Studies have observed significant improvement in apnea-hypopnea and low oxygen saturation among children undergoing rapid maxillary expansion^6^^,^^8^. Majority of the studies have described anatomical abnormalities among individuals, suffering from OSA and its treatment^1^^,^^7^^,^^9^^,^^10^. Few studies have also correlated obstructive OSA and orofacial dysfunction among children^11^. Also, previous review provides a short-term analysis of OSA children and RME^2^^,^^4^^,^^7^.

To bridge this gap, the study reviews the rapid maxillary expansion for obstructive sleep apnea among children. It is also driven due to scarcity of literature on the subject, as indicated by the recent RME review of OSA children by Vale et al.^12^, which is limited to the treatment of AHI normalization. Similarly, the conflicting findings further drive towards the meta and systematic analysis of the effectiveness of RME among OSA children. Such as Felippe et al.^13^ found that RME improved their quality of life, while Langer et al.^14^ showed negative consequence stating that RME had no impact on the breathing of the OSA individual in a short run. To develop a consensus and to provide holistic views, it reviews the studies on rapid maxillary expansion for obstructive sleep apnea among children followed by a meta-analysis on the accessible data. These studies differ based on the objectives and instruments used to portray in-depth information about rapid maxillary expansion to treat obstructive sleep apnea. This review would assist to overcome significant physical and neuro-psychomotor impairment among children, as it needs to be diagnosed and treated early for avoiding chronic problems affecting a child’s neurological development^15^.

## METHODS

### Information sources

The preferred reporting items for systematic reviews and meta-analysis (PRISMA) was practiced. Embase, PubMed, Conference Proceedings Citation Index, Google Scholar, Scopus, The Web of Science and The Cochrane Collaboration Databases were used as information sources.

### Search strategy

Binary operators were comprehensively used in this search strategy. For instance, Sleep *or* Apnea, Maxillary Expansion *and* Apnea, Apnea *or* Hypopnea, Expansion Technique *or* Maxilla, Orthodontic *and* Distraction, Maxillary Expansion *and* Obstructive Sleep. 

### Study selection

The study included pre- and post-rapid maxillary expansion quantitative data reports for children < 18 years old. Similarly, all study designs, languages and published were included. All studies, giving limited or no information about rapid maxillary expansion and obstructive sleep apnea treatment, were excluded (Table 1). Similarly, studies containing only abstracts were excluded too.

**Table 1:** Inclusion and exclusion criteria.

Inclusion Criteria	Exclusion Criteria
Cohort Study	Personal Blogs
Cross-sectional / Retrospective Study	Essays of REM
Case-controlled Study with Comparison or Control Group	Websites and guest post
Studies that report on rapid maxillary expansion and obstructive sleep apnea treatment	Published studies older version (other than 2009 to 2019)
All languages	Incomplete information

Initially, the research first analyzed the abstracts and derived their validity for the concerned subject, i.e., a rapid maxillary expansion for obstructive sleep apnea among children. The reason for the extensive research was to derive and select articles that are consistent with the research area. PICO questions (Participants, Intervention, Comparison and Outcome) were used to narrow down the articles (Table 2).

**Table 2:** PICO question analysis.

Participants	Patients/clinically oriented studies
Intervention	Rapid maxillary expansion
Comparison	Articles that narrate the outcome of the pre- and post-rapid maxillary expansion and examined the obstructive sleep apnea in children
Outcomes	Treatment outcome, prevalence factors and the challenges observed

**Figure 1:**
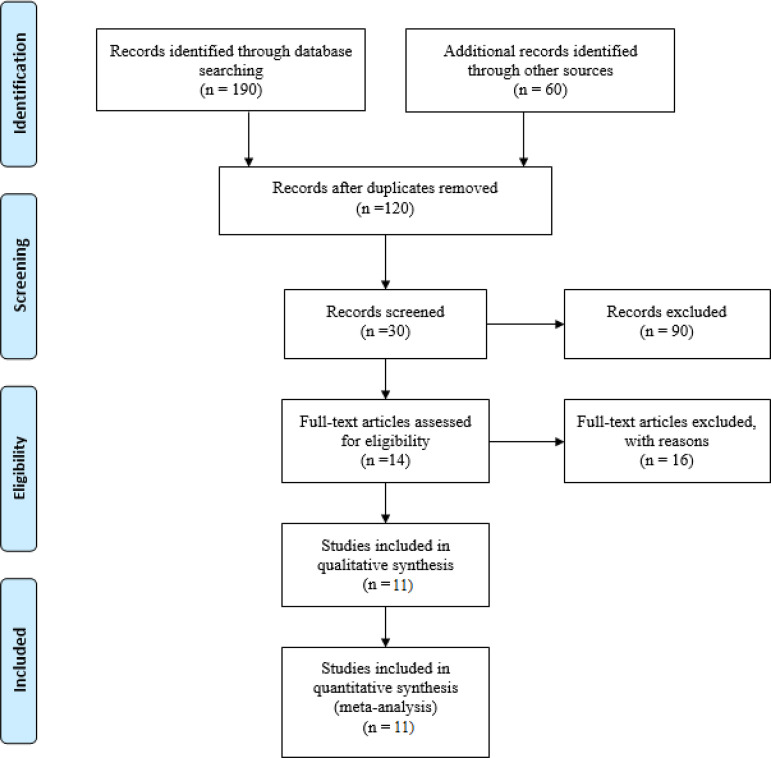
Study procedure.

This review included 11 studies for a comprehensive analysis of rapid maxillary expansion among children with obstructive sleep apnea. Figure 1 presents the procedure for selection of the relevant 11 studies.

### Outcome measures

Mean oxygen saturation and AHI apnea index was included as specific outcome measures. The AHI apnea index is based on the following criteria:

None/Minimal: AHI < 5 per hour;

Mild: AHI ≥ 5, but < 15 per hour;

Moderate: AHI ≥ 15, but < 30 per hour;

Severe: AHI ≥ 30 per hour.

### Data collection procedure

The researcher has systematically and independently searched the accessible literature from 2009 through 2019. 

### Quality evaluation

The National Institute for Health and Clinical Excellence (NICE) tool was used for quality evaluation. The grading of the selected studies was based on high quality if ≥ 6 NICE criteria were met.

### Summary measures

The mean (M), standard deviation (SD), 95% confidence interval (CIs), mean difference, percentage change and standardized mean difference were evaluated for pre-and post-rapid maxillary expansion.

### Statistical analyses

Statistical analyses were performed using Excel for calculating mean, standard deviation and 95% confidence interval. STATA was used for calculating the mean difference and standardized mean difference.

## RESULTS

### Study Selection

The study partitioned 157 study titles and abstracts, where 78 potentially relevant studies were extracted for explicit review after searching through the databases. Similarly, five studies were excluded due to cumulative and duplicate data, while six studies had unique data and met the inclusion criteria.

### Study assessment

Three studies were identified for their high quality (≥ 6 NICE criteria met) using the NICE quality assessment tool, while two studies met lower quality through assessment tool (≤ 6 NICE criteria met) (Table 3).

**Table 3:** General characteristics and quality criteria.

Quality Assessment									
Study Design	Outcomes analyzed	1	2	3	4	5	6	7	8
Vale et al.^12^ Prospective case control.	AHI, MSAT	No	Yes	Yes	Yes	Yes	Yes	Yes	Yes
Borgström^19^ Prospective case control	AHI, MSAT	No	Yes	Yes	Yes	Yes	Yes	Yes	Yes
Fransson, Kowalczyk and Isacsson^20^ retrospective case control	AHI	No	Yes	No	Yes	Yes	Yes	Yes	No
Detailleur et al.^16^ Retrospective case control	AHI, MSAT	No	Yes	Yes	Yes	No	Yes	Yes	Yes
Ng et al.^17^ Prospective case control	AHI	No	Yes	Yes	Yes	Yes	No	Yes	Yes
Melo et al.^10^ retrospective case control	AHI, MSAT	No	Yes	Yes	Yes	Yes	Yes	Yes	No

### Outcomes

Seven studies with 94 unique patients met criteria (age = 6.4 ± 1.7 years). Follow-up duration was assessed to analyze the data such as ≤ three years (94 patients) and > 3 years (52 patients). The studies included in the meta-analysis met the criteria and provided mean and standard deviation for respective patients. For instance, Melo et al.^10^ reported means and standard deviations for 29 patients (age = 6.3 ± 1.5 years). For Vale et al.^12^ data were obtained for 20 patients that reported means and standard deviations for rapid maxillary expansion. Two studies have reported complications and indicated that there were no complications among patients who underwent rapid maxillary expansion^15^^,^^16^.

### Apnea-hypopnea index and mean oxygen saturation

The pre- and post-rapid maxillary expansion for apnea-hypopnea index was reduced from MD ± SD of 12.05 ± 5.62 to 2.6 ± 1.96 for > 3 years follow-up. The reduction was found to be 77% after > 3 years follow-up of rapid maxillary expansion. Similarly, the pre- and post-rapid maxillary expansion reduced from an MD ± SD of 8.46 ± 7.82 to 3.2 ± 2.62, showing a reduction of 73% after ≤ 3 years follow-up. For mean oxygen saturation, the pre- and post-rapid maxillary expansion enhanced from MD ± SD of 57.4 ± 10.2% to 57.9 ± 10.55% for > 3 years follow-up in 52 patients. Similarly, the pre- and post-rapid maxillary expansion enhanced from MD ± SD of 94.46 ± 2.1% to 95.6 ± 1.12% after ≤ 3 years follow-up (Table 4).

**Table 4:** Demographic analysis before and after rapid maxillary expansion.

Study	No	Age, yr.	BMI, kg/m^2^	Pre-RME AHI	Post-RME AHI	Pre-RME MSAT	Post-RME MSAT
> 3 years follow-up							
Detailleur et al.^16^ retrospective case control	27	21.1 ± 7.24	23.6 ± 2.72	21.1 ± 7.24	2.9 ± 2.42	23.8 ± 18.4	23.2 ± 18.2
Ng et al.^17^ Prospective case control	25	8.9 ± 0.8	15.8 ± 2.0	3 ± 4	2.3 ± 1.5	91 ± 2	92.6 ± 2.9
Total	52	15 ± 4.02	19.7 ± 2.36	12.05 ± 5.62	2.6 ± 1.96	57.4 ± 10.2	57.9 ± 10.55
≤ 3 years follow-up							
Melo et al.^10^ retrospective case control	29	6.3 ± 1.5	18.5 ± 3.7	5.5 ± 5.9	2.3 ± 1.3	96.3 ± 1.3	96.1 ± 0.7
Vale et al.^12^ Prospective case control	20	7.5 ± 1.7	17.8 ± 2.1	5.9 ± 6.4	2.9 ± 1.9	95 ± 5	97 ± 1.3
Borgström^19^ Prospective case control	13	6.2 ± 1.9	16.7 ± 3.4	6.0 ± 4.3	2.7 ± 1.3	95.3 ± 1.5	97.5 ± 0.6
Fransson, Kowalczyk and Isacsson^20^ retrospective case control	9	6.5 ± 2.1	18.0 ± 3.3	17.2 ± 19	5.2 ± 6.1	94 ± 1.4	95.5 ± 2.3
Total	94	6.4 ± 1.7	14.2 ± 2.5	8.46 ± 7.82	3.2 ± 2.62	94.46 ± 2.1	95.6 ± 1.12

Random effects model calculation for rapid maxillary expansion

The random effects model calculation for pre- and post-rapid maxillary expansion is determined through mean and standardized mean difference. The mean difference reported by Detailleur et al.^16^ for 27 patients was -2.87 (95% CI = -4.83 to -0.89) for the apnea-hypopnea index. Similarly, the mean difference reported by Ng et al.^17^ for 25 patients was -0.39 (95% CI = -0.89 to 0.09) for the apnea-hypopnea index. The mean difference reported by Melo et al.^10^ for 27 patients was -3.47 (95% CI = -4.15 to -2.79) for the apnea-hypopnea index. Similarly, the mean difference reported by Vale et al.^12^ for 20 patients was -3.17 (95% CI = -5.79 to 0.55) for the apnea-hypopnea index. The mean difference reported by Bower and Gungor^18^ for 23 patients was -2.55 (95% CI = -4.03 to -1.11) for the apnea-hypopnea index. Similarly, the mean difference reported by Borgström^19^ for 13 patients was -6.59 (95% CI = -8.93 to -4.32) for the apnea-hypopnea index. Fransson et al.^20^ reported a mean difference for 9 patients as -4.59 (95% CI = -7.59 to -1.29) (Table 5).

**Table 5:** Mean difference and standardized mean difference before and after rapid maxillary expansion.

Study	Post-Rapid Maxillary Expansion			Pre-Rapid Maxillary Expansion			Mean Difference
	Mean	SD	Total	Mean	SD	Total	95% CI
Detailleur et al.^16^	2.5	2	27	5.5	3.3	27	-2.87 [-4.83, -0.89]
Ng et al.^17^	4.6	0.9	25	5.1	0.7	25	-0.39 [-0.89, 0.09]
Melo et al.^10^	1.3	0.4	29	4.9	1.2	29	-3.47 [-4.15, -2.79]
Vale et al.^15^	2.4	1.3	20	5.6	5.8	20	-3.17 [-5.79, -.55]
Borgström^18^	5.3	5.3	13	11.9	4.5	13	-6.59 [-8.93, -4.32]
Fransson et al.^20^	2.1	1.3	9	6.5	4.6	9	-4.59 [-7.59, -1.29]
Total (95% CI)			146			146	

### Systematic Analysis

The systematic analysis was conducted to present new and comprehensive insights into the researched area. 11 studies were included in the systematic analysis. The first study included is of Campbell et al.^21^ which assessed the use of RME among Cleft palate with or without cleft lip (CP/L) individuals with obstructive sleep apnea. 24 children aged 6 to 12 years were included in the study. Pediatric sleep questionnaires (PSQ) were used for analyzing the pre and post RME results of the patients, which revealed that majority were at an increased risk of OSA before the treatment. However, prior to the treatment, the symptoms were found to have improved irrespective of the cleft lip. 

REM was also used by Fastuca et al.^22^ for examining the changes in the patients’ airway volume. It analyzed the correlation that existed between the changes in the morphological and respiratory functions that occur as a result of rapid maxillary expansion. It examined 15 patients using computed tomography and polysomnography examination before the rapid maxillary expansion and after maxillary expander with an interval of 12 months later. The volume analysis showed that the airway volume was high, along with the oxygen saturation level. The modifications made through the treatment were also significant (*p*<0.05). The results showed that the use of rapid maxillary expansion improved the patients’ outcomes and led to an improvement in their conditions. Such as many of the patients reported that the air volumes were more reduced both middle and low.

Guilleminault et al.^23^ conducted a power analysis to examine the enlarged tonsils problems among the children and how it impacted their response. 31 children with OSA were included in the study with clinical symptoms and polysomnography. The participants also reported the presence of both narrow maxillary complex as well as enlarged tonsils. A randomized control trial was used in which the first group had surgery following orthodontics while another group first had orthodontics and then surgery. The results showed that no child in group 1 presented normal outcomes except for one, while improvements were found in children of group 2 for clinical symptoms and polysomnography. It emphasized that for improving the study results, a more effective clinical scale should be determined, which helps in appropriate sequencing of the treatments.

Taddei et al.^24^ examined the impact of the rapid maxillary expansion and the mandibular advancement on the children upper airway. Experimental study design was used, and 30 children with Marfan’s syndrome were included while the control group had untreated children. Data was collected at different points; such as before the treatment, after rapid maxillary expansion, and then after mandibular advancement, which was same for the control group. The results should reflect that oxygen desaturation and apnea-hypopnea was substantially high for the experimental group while for the control group, these were low. The findings showed that rapid maxillary expansion at an early stage and mandibular advancement help in improving the airway patency among children with Marfan’s syndrome. It also suggested that the routine check-up should be held for the treatments, particularly for cases with class II malocclusions and prevention against obstructive sleep apnea.

Likewise, Buccheri et al.^25^ also assessed the efficiency of the Rapid Maxillary Expander in OSAS young patients through measurement of the cardio-respiratory monitoring parameters. The study was conducted on 11 young subjects where all were treated using RME. Cardiorespiratory monitoring (8-channel Polymesam) was held at an interval of 12 months. The results showed improvement in the clinical symptoms; which comprise of reduced sleep apnea and soring among the children. Overall, the study established the efficiency of the RME for OSA young patients. It concludes that it serves as an effective option for orthopedic-orthodontic treatment.

Pirelli et al.^26^ also used the RME therapy for assessing its skeletal effect while with anchors such as teeth in OSA children. This was achieved using the low-dose computed tomography on maxillary base width, midpalatal suture opening, first molar angulation, nasal cavities width and pterygoid processes distance. It included 14 children and used a 16-row MSCT scanner before and after the treatment followed by a program for Dentascan reconstruction. The results showed that CT is rigid in operations for the analysis.

Similarly, Kim^27^ evaluated the effectiveness of orthodontic treatment using rapid maxillary expansion (RME) concerning its management of pediatric obstructive sleep apnea (OSA). A case of 11-year-old boy was considered who experienced severe pediatric. The polysomnographic findings showed that orthodontic treatment following RME was effective in OSA management among the patients.

Helal et al.^28^ research examined the perception of the parents’ concerning the change in the behavior following their children treatment with RME and the factors linked with sleep quality and fatigue. 91 children aged from 5 to 13 years were included who had a deficiency of transverse maxillary and no major disease. The post and pre RME outcomes showed improvements where a decrease in dry mouth rate, snoring time and heavy breathing was found. Overall, the parents showed that RME post-treatment showed great improvement in the children’s behavior, which resulted in improved sleep quality and breathing.

Pirelli et al.^29^ prospectively assessed the effect of RME among the children with OSA in the long run. It included 31 children that had OSA, isolated maxillary narrowing and lacked enlarged adenotonsillar. The follow-up time in the study was one-year, where later in the teenage, these children underwent clinical reevaluation and repeated polysomnography (PSG). Lastly, the participants were evaluated using computerized tomographic (CT) imaging and the results were compared among both pre- and post-test. Normal results were observed over time in terms of their clinical evaluations along with their PSG findings. The maxillary base width was found to be stable over time. It showed the effectiveness of the RME treatment in the long-run.

Villa et al.^7^ used RME for evaluating the orthodontic treatment positive outcomes for the sleep disorder breathing among 14 OSA children with 36 months of follow-up. The results revealed a decrease in the apnea hypopnea index (AHI) as well as clinical symptoms at the treatment conclusion. However, at the interval of 24 months, no changes were found. Primarily, it concludes that RME treatment can be used for deriving effective outcomes of the children with OSAS and malocclusion; however, its effects are observed at the end of the treatment. Likewise, Ashok et al.^30^ also examined the RME impact on the sleep characteristics of the patients among children aged between 8 to 13 years. It cemented bonded rapid maxillary expander in its sample, where intermolar distance was measured at the start of the treatment and the end, with a follow-up period of 3 months. The average of the sleep characteristics was computed using the nonparametric Friedman test, while the intermolar width was compared using the Wilcoxon signed ranks. The findings concluded that sleep efficiency improved after the treatment, with an improvement in the sleeping time. It also led to an improvement in the posterior crossbites, coordination of the mandibular dental arch and maxillary.

## DISCUSSION

This study examined three main findings after reviewing international studies for rapid maxillary expansion in treating obstructive sleep apnea. Firstly, the findings have indicated that apnea hypopnea index was enhanced after rapid maxillary expansion in children with obstructive sleep apnea. Secondly, there is a 73% enhancement in the apnea hypopnea index in children with obstructive sleep apnea after ≤ 3 years follow-up. Thirdly, there was 77% enhancement in the apnea hypopnea index in children with obstructive sleep apnea after > 3 years follow-up. The effectiveness of rapid maxillary expansion in the treatment of obstructive sleep apnea is studied by Vale et al.^12^ and stated an overall apnea hypopnea index after rapid maxillary expansion therapy in 18 children. Some children were treated with non-surgical treatment with continuous positive airway pressure. The evaluation and surgical treatment of pediatric obstructive sleep apnea are reported by Borgström^19^ and stated that obstructive sleep apnea was reduced in 79 children after treating them with adenotonsillectomy and rapid maxillary expansion.

Another important finding examined was the improvement in mean oxygen saturation for > 3 years and ≤ 3 years follow-up. The selected studies have comprehensively reported the improvement in mean oxygen saturation after treating children with rapid maxillary expansion. Fransson et al.^20^ have measured and evaluated 74 patients with obstructive sleep apnea, using a mandibular protruding device. The results revealed improvement in the mean oxygen saturation with infra-occlusion and mesio-occlusion after being treated with mandibular protruding device. Similarly, Detailleur et al.^16^ documented the impact of maxillary expansion in children with sleep disorder symptoms. It demonstrated improvements in apnea hypopnea index and mean oxygen saturation in 27 children after treating with rapid maxillary expansion. Moreover, small cephalometric changes were observed between both groups.

Based on discussion above, it is considered that children should be treated with rapid maxillary expansion and should be referred to an orthodontist or a pediatric dentist, a dental specialist, or a multidisciplinary surgery. The study recommends that practitioners need to consider the RME treatment for the orofacial abnormalities among children, including adenoids and maxillary-mandibular for sleep disorder breathing. Moreover, each case should be comprehensively studied where the clinical focus can vary based on the anatomical problems experienced by the children. Such as large tonsils can cover the skeletal structure involvement, while the involvement of the skeletal structure can mask the role of the soft tissues. The practitioners need to comprehensively evaluate the head to neck examination of the children to identify the presence of malocclusion among the children that can cause sleep-disordered breathing and determine whether the case should be referred to sleep specialist or not.

## CONCLUSION

The study has reported enhancements in apnea hypopnea index and mean oxygen saturation in children with obstructive sleep apnea, who underwent rapid maxillary expansion, specifically in the short-term and long-term follow-up. The effect and spontaneous resolution of obstructive sleep apnea are determined through these findings. The frequency of maxillary expansion is not described comprehensively through any standardized nomenclature. The selected articles vary in the context of measurements and therapies used to treat obstructive sleep apnea children. The articles included in this meta-analysis reported different outcomes based on different inclusion or exclusion criteria and different patient populations. It is difficult to sketch core conclusions due to these limitations from baseline to follow-up parameters. All the selected articles are based on case series and case reports regardless of two randomized trials. Moreover, snoring and quality of life after rapid maxillary expansion were not reviewed, and thus, allow future researchers to investigate these characteristics along with current ones.

## Figures and Tables

**Table 6:** Characteristics of the selected studies.

Name of the Author	Year of Publication	Study Title	Type of Study
Campbell^21^	2018	Rapid maxillary expansion and protraction alleviates obstructive sleep apnea in non-syndromic children with cleft palate (doctoral dissertation, UCSF).	Cross-sectional
Campbell^21^	2018	Rapid maxillary expansion and protraction alleviates obstructive sleep apnea in non-syndromic children with cleft palate (doctoral dissertation, UCSF).	Cross-sectional
Guilleminault et al.^23^	2011	Adeno-tonsillectomy and rapid maxillary distraction in pre-pubertal children, a pilot study.	Pilot Study
Taddei et al.^24^	2015	Effects of rapid maxillary expansion and mandibular advancement on upper airways in Marfan's syndrome children: a home sleep study and cephalometric evaluation.	Experiment
Buccheri et al.^25^	2017	Rapid maxillary expansion in obstructive sleep apnea in young patients: Cardiorespiratory monitoring.	Cross-sectional
Pirelli et al.^26^	2012	Rapid maxillary expansion before and after adenotonsillectomy in children with obstructive sleep apnea.	Experimental study
Kim^27^	2014	Orthodontic treatment with rapid maxillary expansion for treating a boy with severe obstructive sleep apnea.	Case Study
Helal^28^	2019	Parents' perceptions of breathing pattern changes, sleep quality, and fatigue in children after rapid maxillary expansion: a survey and case series study.	Case series study
Pirelli et al.^29^	2015	Rapid maxillary expansion (RME) for pediatric obstructive sleep apnea: a 12-year follow-up.	Prospective
Villa et al.^7^	2011	Efficacy of rapid maxillary expansion in children with obstructive sleep apnea syndrome: 36 months of follow-up. Sleep and Breathing.	Experimental study
Villa et al.^7^	2011	Efficacy of rapid maxillary expansion in children with obstructive sleep apnea syndrome: 36 months of follow-up. Sleep and Breathing.	Experimental study

**Table 7:** Characterizing studies based on their core aim.

Study	Aim	Patients
Campbell^21^	The study aimed to assess the use of rapid maxillary expansion among individuals with or without cleft palate.	24 Patients aged between 6-12 years were included. Most of the patients demonstrated the increasing risk factors for obstructive sleep apnea.
Fastuca et al.^22^	The examinations regarding the changes in the airway volume of patients by using the rapid maxillary expansion were undertaken in the given study. Correlation between the morphological and respiratory functions were examined.	Patients indicated a high volume of the airway with the saturation levels of oxygen. Furthermore, the usage of the rapid maxillary expansion provided significant outcomes through valuable improvements.
Guilleminault et al.^23^	The study conducted a power analysis to examine the enlarged tonsils problems among the children and how it impacted their response.	None of the children in group one presented normal outcomes except for one, while improvements were found in group 2 of the children for clinical symptoms and polysomnography. Recommendations regarding the study, followed by a clinical scale, were made.
Taddei^24^	The study examined the impact of the rapid maxillary expansion and the mandibular advancement on the children upper airway.	Oxygen desaturation and apnea-hypopnea were substantially high for the experimental group, while for the control group these were low. Also, rapid maxillary expansion at an early stage and mandibular advancement helps in improving the airway patency among children with Marfan's syndrome.
Buccheri et al.^25^	The study assessed the efficiency of the Rapid Maxillary Expander in OSAS young patients through measurement of the cardio-respiratory monitoring parameters.	The study established the efficiency of the RME for the OSA young patients.
Pirelli et al.^26^	RME therapy was used to assess the skeletal effect of the rapid maxillary expansion with anchors suc h as teeth among OSA children.	The results indicated that CT is rigid in operations for the analysis of the skeletal effect of rapid maxillary expansion.
Kim^27^	The study evaluated the effectiveness of orthodontic treatment by using rapid maxillary expansion (RME) concerning its management of pediatric obstructive sleep apnea (OSA).	Findings indicated the effectiveness of RME in OSA management among the selected patients.
Helal et al.^28^	Examined the perception of parents' concerning the behavioral changes among children involved in treatment with RME. It further identified factors linked to sleep quality and fatigue.	Results demonstrated that RME post-treatment is a significant improvement in the children' behavior, which resulted in improved sleep quality and breathing.
Pirelli et al.^29^	The assessment regarding the effect of RME among children with OSA was held in this study.	Normal results were provided in the given patients referring to the stable maxillary width and clinical evaluations.
Villa et al.^7^	Evaluation regarding the positive outcomes of the orthodontic treatment for the sleep disorder breathing among 14 OSA children with 36 months of follow-up has conducted the study. The evaluation was done through RME.	Results demonstrated a valuable decrease in the apnea hypopnea index (AHI) along with the observation of the clinical symptoms.
Ashok et al.^30^	Impact of RME on sleep characteristics of the patients that include children aged between 8 to 13 years was observed in the study.	Improvements in the form of sleep efficiency, sleeping time, posterior crossbites, coordination of the mandibular dental arch and maxillary were found.
